# New Appliances and Things Medical

**Published:** 1894-11-24

**Authors:** 


### NEW APPLIANCES AND THINGS MEDICAL.

[We shall be glad to receive, at our Office, 428, Strand, London, W.C., from the manufacturers, specimens of all new preparations and drugs which may be brought out from time to time.]

### SCOTT AND SCOTT'S "COCOANA."

(Weston, Hunt, and Co., 39, Seething Lane, Great Tower Street, London, E.C.)

This new preparation is a mixture of the essential constituents of the banana with pure cocoa essence and other dietetic ingredients. Amongst these latter we have found a considerable proportion of cane sugar and starch. In appearance it resembles many of the cocoatinas and chocolate powders at present in the market, but the judicious admixture of the banana flour has given to it a most agreeable flavour so that the addition of boiling water or milk produces a beverage which is not so thick and heavy as that yielded by the ordinary cocoa preparations. The proprietors seem to have taken advantage of the well-known digestive properties of the banana to make the cocoa of much greater benefit to dyspeptics, invalids, and children. The quantity of fat which we have found in this preparation is less than that which is usually met with in commercial cocoas, but this is, of course, to be accounted for by the fact that it is admixed with the banana flour. There is 1·9 per cent. of nitrogen present, and the amount of ash was 4·33 per cent. in the sample sent to us for examination. The growing popularity of cocoa has no doubt led the proprietors to place this preparation on the market at the present time, and we hope that their efforts will be successful as, in our opinion, it has a wide field of usefulness.

